# A prospective longitudinal approach to examine the association between social position in childhood, adolescence, and adulthood with the control of hypertension during adulthood

**DOI:** 10.3389/fpubh.2024.1296593

**Published:** 2024-04-12

**Authors:** Susana Barradas, Diego I. Lucumi, Graciela Mentz, Diana Maria Agudelo

**Affiliations:** ^1^School of Social and Human Sciences, Universidad Externado de Colombia, Bogotá, Colombia; ^2^Alberto Lleras Camargo School of Government, Universidad de los Andes, Bogotá, Colombia; ^3^Statistician Lead, Anethesiology Department, Medical School, University of Michigan, Ann Arbor, MI, United States; ^4^Psychology Department, School of Social Sciences, Universidad de los Andes, Bogotá, Colombia

**Keywords:** life course social position, hypertension control, socioeconomic position, social determinants of health, hypertension

## Abstract

**Introduction:**

Hypertension is one of the main concerns in public health, since it is related with increased morbidity, and potential years of life lost in addition to loss of quality of life. This study aimed to assess: (1) the distribution of indicators of life course SEP in a cohort of Colombian patients with hypertension and (2) to assess the association of life course SEP and control of hypertension among this cohort of patients.

**Methods:**

Data were obtained using the baseline survey of 258 patients from the Social Determinants and Inequities in the Control of Blood Hypertension Program (ProDSICHA). Mother occupation and housing conditions were measured with the Event History Calendar. Mother educational level was measured with the questionnaire developed by the Project on Ethnicity and Race in Latin America (PERLA). Socioeconomic position during adulthood was measured using education, occupation, and income level based in the MacArthur Network.

**Results:**

The group with a higher lifelong social position and the group of lower lifelong social position showed better control of hypertension (OR = 1.21; *p* <0.05; OR = 1.33; *p* < .05, respectively) compared to those whose social position throughout life varied the most. No statistical differences were found in the relations between single lifetime social position variables, and hypertension control in the three time points analyzed.

**Discussion:**

These findings warrant further research to deeper our understanding on the role of a multidimensional and cumulative approach of social position in hypertension control.

## Introduction

Worldwide, hypertension has been considered one of the main public health problems (1). This condition is the most important modifiable risk factor for cardiovascular disease, the leading cause of death globally (1). In addition, hypertension negatively impacts patient’s quality of life (2, 3). Globally, evidence shows a stable age-standardized prevalence of hypertension among both men and women when comparing 1990 with 2019. This stability is a net effect of a decrease in high-income countries and an increase in some low- and middle-income countries (LMICs), including some in Latin America (4). Despite the consistent worldwide prevalence, the total count of individuals aged 30–79 years diagnosed with hypertension saw a two-fold increase. In 1990, there were 331 million women and 317 million men affected, and by 2019, this number had risen to 626 million women and 652 million men, primarily attributed to population growth and aging (4). Regarding treatment and control in LMIC during the same period, both improved in most countries since 1990, but the proportion of those treated who achieved control varied by more than four times across countries. However, akin to high-income countries, some in Latin America and the Caribbean demonstrated a closer alignment between treatment and control rates (4).

In Colombia, hypertension prevalence is between 24 and 31.1% (4, 5). In addition, in the country, 76.3% of the women with hypertension are aware of their status, 63.9% are being treated, and 41% of hypertension in these patients is actually controlled (4). In men, data showed that 60.3% are aware of their health situation, 45.6% are being treated, and 24% have their hypertension controlled (4). Furthermore, reports about Colombia showed a high prevalence of risk factors associated with hypertension as follows: 56.5% of the population is overweight, 51.1% is sedentary (6), 23% smokes, and 61% has dyslipidemia (7), which place the population at a higher risk of coming to have hypertension.

Although hypertension has a significant impact on mortality and morbidity, there is a paucity of research on social determinants associated with hypertension control. Socioeconomic position (SEP) during the life course has been related to disadvantage processes that have implications for health outcomes across the lifespan (8, 9). The life course approach posits that “combination, accumulation, and/or interaction of the social environments and biological insults experienced throughout the life course impact current and future events, environments, and health conditions and thus ultimately impact adult health” [(10), p. 2]. To study the influence of SEP on cardiovascular research, the latent effects, pathways, social mobility, and cumulative life course models have been proposed. The operation of the hypothesis proposed by one or more of these models helps to explain the role of socioeconomic advantage or disadvantage in shaping cardiovascular risks and outcomes (10).

### Effects of SEP on health

SEP has been related to health in general in such a way that the lower the occupied position in the social ladder, the worse the health outcomes in the individuals. Different studies have shown how socioeconomic disadvantage leads to poorer health outcomes [e.g., Shea et al. (9)], higher mortality [e.g., Brown et al. (11) and Stringhini et al. (12)], and a worse quality of life in general (13, 14). Other studies have shown that subjective perception of socioeconomic status also has a relation with health status in adults [e.g., Demakakos et al. (15) and Richards et al. (16)].

There have been different efforts to conceptualize and explain how SEP and inequality in general can *accumulate* and affect individuals’ health and mortality. One such example is the *cumulative inequality theory*, which places macrostructural elements as the basis of health inequity (17). Furthermore, it specifies how these inequities are expressed “over the life course via demographic and developmental processes, and that personal trajectories are shaped by the accumulation of risk, available resources, perceived trajectories, and human agency” [(17), p. 334]. Two important premises of this theory are that (1) inequality accumulates throughout life and (2) the life situation in childhood allows us to explain functioning levels and general wellbeing in adulthood (17). Also relevant is the idea that social systems are the ones that generate inequality (17, 18).

Another important piece of the puzzle that allows us to deepen this topic is the *accumulation of risk models*, which also suggest that “childhood circumstances set individuals on diverse social, economic, and behavioral trajectories that in turn affect health” [(19), p.139]. In this sense, early social disadvantages begin a chain of negative influences that are later reflected in worse health and premature death (19, 20). From a structuralist approach, there are several influences that have been considered to affect health and which are deeply linked with SEP. Some examples are diet, housing, working conditions, exposure to air pollution, and access to health services (21). Another approach is related to the psychological stress that could be present in deprived situations. It has been pointed out that the perceived stress that individuals might experience in adverse psychological or material situations can have a negative impact on health (22).

### SEP in childhood and health outcomes in adulthood

Adult health status has been related to social circumstances across the life course, particularly with socioeconomic conditions (e.g., 23–25). For example, adverse childhood experiences have been related to long-term physical health consequences [e.g., Monnat and Chandler (26) and Nurius et al. (27)]. Furthermore, results from a systematic review showed that childhood and adolescent socioeconomic circumstances were determinants for adulthood’s greater risk for cardiovascular disease (28). Moreover, some studies have shown that this effect is independent of SEP in adulthood (29).

In this regard, evidence also shows a relationship between SEP during childhood and high adult blood pressure (23, 30) which accounts for the importance of early life in the development of hypertension (31). Nevertheless, there is also contrary evidence that reports no relationship between those variables along the individuals’ life course (29). It is worth mentioning that the studies that found no relationship used mostly correlational and prospective designs (trend studies), while studies that found an association between childhood SEP and adult blood pressure were prospective cohort studies (29).

A way of approaching methodologically and theoretically to life course and particularly childhood and adolescence SEP indicators is through some parents’ variables [e.g., Galobardes et al. (32)]. For instance, studies showed that housing conditions during childhood and adolescence are associated with poor health outcomes such as respiratory infections, asthma, lead poisoning, injuries, and mental health (33). In addition, parent’s education influences health in older ages. A study found that the mother’s education level (but not the father’s) was associated with self-rated adult health at ages 33 and 42 years: the higher the educational level achieved by the mother, the better health status perceived (34). Moreover, a study in rural Uganda showed that the mother’s education level (but not the father’s) was an independent predictor for better health and nutrition among infants and young children (35). Another study found that the higher the level of a mother’s education, the higher the direct effect on child health. In this study, no statistical differences were found regarding the father’s education level (36). Another important reference for our study is the Jackson Heart Study (37), whose results showed that a higher mother’s education was protective of hypertension prevalence in adulthood. Thus, these studies suggest that a mother’s education plays a key role in predicting health outcomes in her offspring.

Regarding parents’ occupation, the study by Pinilla et al. (38) showed that parental occupation is associated with the probability of reporting good health in adults ages. The study also reported that the father’s occupation effect was more important than the mother’s. In this same study, results showed that children of those women in top occupations show the highest probability of having good health.

**Figure 1:**
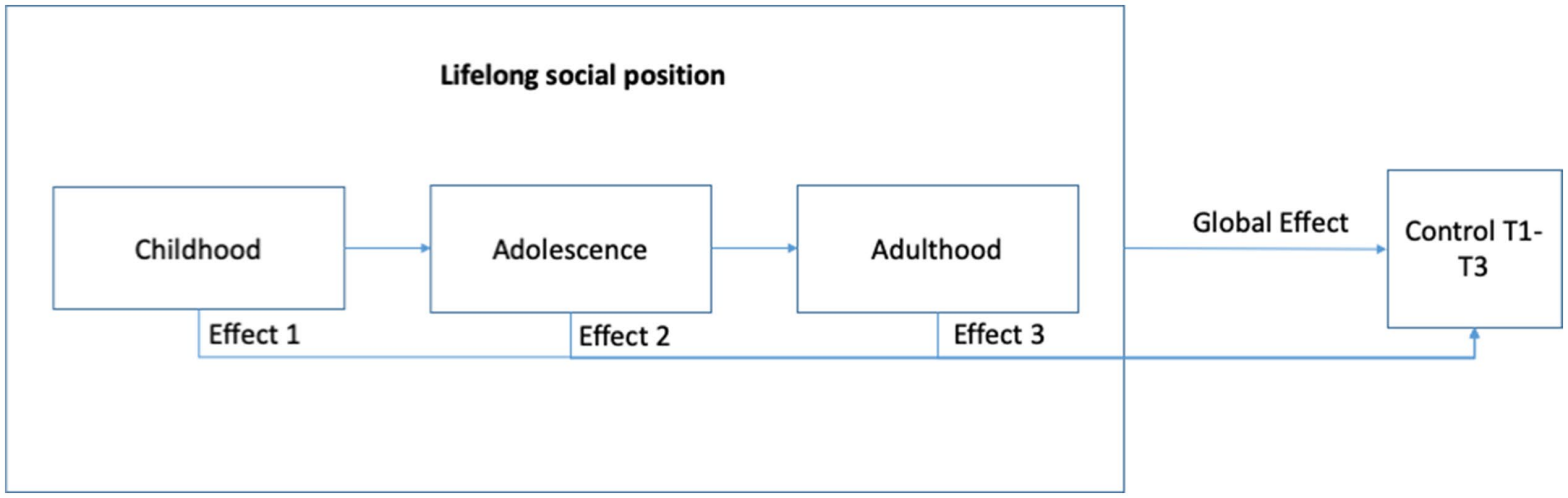
Research model with variables in the study.

In Latin America, there is a paucity of research about the role of SEP and cardiovascular risk factors during the life course. This is problematic, as SEP throughout one’s life may contribute to understanding the cardiovascular profile within and between Latin American countries. In addition, to the best of our knowledge, there is no research in Colombia studying the role of life course SEP on hypertension control in adulthood. Since hypertension remains a public health problem in low- and middle-income countries (39), including those in Latin America, it is important to deepen our understanding of the influence of life course SEP variables on this condition, particularly in the context of Colombia, one of the most economically unequal countries in the world. To contribute to the literature on this subject, the present study aims to test the model shown in Figure 1.

Considering the above conceptualization, the aims and hypothesis of this study are as follows:

Aim 1: The first objective of this study is to assess the distribution of indicators of life course SEP in a cohort of Colombian patients with hypertension.
Aim 2: The second objective of this study is to assess the association of life course SEP and control of hypertension among this cohort of patients.

*Hypothesis 1*: Single-life course SEP indicators (childhood, adolescence, and adulthood) are directly associated with hypertension control.

*Hypothesis 2*: Combined life course SEP indicators are directly associated with hypertension control.

## Method

### Data

Data for this study came from the Social Determinants and Inequities in the Control of Hypertension Program (ProDSICHA for its Spanish acronym) project. This was a prospective longitudinal study that included hypertension patients from 45 to 70 years who participated in hypertension programs in Colombia. ProDSICHA study aimed at furthering our understanding of the effects of social determinants of hypertension control in three Colombian cities. There were three data collection periods for this project. The assessments took place between 2015 and 2018. This study was approved by the Universidad de los Andes Ethics Committee (Act number 531 of 2015).

### Population and sample

We designed the sample using a multi-stage sampling strategy (cities, institutions, and participants). In phase one, we selected three cities using heterogeneous criteria to examine the variations and similarities that the phenomenon may present in different contexts (40). We selected Bogotá, Medellín, and Quibdó, considering differences in poverty levels, health service infrastructure, and city size. In phase two, we selected health-promoting entities (HPEs) that had established a hypertension control program through their network of healthcare providers. In some HPEs, the majority of affiliates belonged to the subsidized regime, which covers people without the ability to pay and the most vulnerable population in the country classified according to the Beneficiary Selection System, while in others, the majority belonged to the contributory regime, which requires a monthly contribution from affiliated persons and covers employees under labor contracts in the public or private sector, pensioners, and independent workers with the ability to pay, as well as the beneficiaries of the affiliate.

Other selection criteria for the HPEs included coverage equal to or greater than the pre-determined sample size of each city, the existence in these institutions of an updated record of their service users, and the institution’s agreement to participate in the study. In phase three, we selected the participants from a list obtained from each HEP. A stratified probability sampling strategy was used based on age and gender distribution.

The inclusion criteria for the participants were as follows: adults aged between 45 and 70 years with diagnosed hypertension, participants of ProDSICHA at the selected institution, and managed as outpatients. Participants with cognitive, neurological, psychiatric, or motor impairments that hinder response capacity to psychological tests were excluded. We used data from the three waves of the ProDSICHA project.

Participants were contacted to respond to the questionnaire in the three waves of the study. If, after three unanswered calls, it was not possible to contact the participant, the reference contact provided by the person was called (if this contact was not successful after three call attempts, the participant was withdrawn from the study). In each city and wave, the interviews and measurements were conducted at the affiliated university campus. All participants were reimbursed for transportation from their residence to each campus.

### Instruments and measurements

#### SEP during childhood and adolescence

##### Mother’s occupation

In the Event History Calendar developed, we included the questions *“What was your mother’s occupation when you were 7 years old?”* and *“What was your mother’s occupation when you were 14 years old?.”* Response categoric options were “employee,” “freelance,” “unemployed,” “student,” “employee and student,” “unpaid homework,” “housewife or husband,” “retired,” “absent,” “passed away,” “do not know,” “do not answer,” and “does not apply.”

##### Mother’s educational level

We used the questionnaire developed by the Project on Ethnicity and Race in Latin America (PERLA) (41), specifically, the question “*What is or was the highest educational level completed by your mother?*.” The response options were categoric and varied between “none” and “postgraduate.”

##### Housing conditions

To assess objective housing conditions during childhood and adolescence, an Event History Calendar was developed for this study. Specifically, we used the questions: *“What material was your home mainly when you were 7 years old?”* and *“What material was your home mainly when you were 14 years old?.”* The response options were six categoric items: “cement mainly,” “wood mainly,” “other,” “do not know,” “do not answer,” and “does not apply.”

#### SEP during adulthood

To assess socioeconomic position during adulthood, we used three variables based on the McArthur Network: *education, occupation*, and *income level*. To measure *education*, we asked participants what level of education they had reached. Response options were elementary school, high school, elementary school incomplete or no studies, technical or technological studies, university degree, and a postgraduate degree. For the purpose of statistical analysis, we categorize the variable into three options: “less that elementary school,” “high school,” and “more that high school.”

To measure *occupation*, we asked participants which options best described their work. Response options were student, retired, housekeeper, employee and student, independent worker, employed, and unemployed. In the analysis, the variable was categorized into “employed,” “unemployed,” “housekeeper,” and “retired.”

Finally, we measured the *income level* by asking participants “¿How much did you earn last month?.” Response options vary between “Less than a current legal minimum wage” and “More than 16 current legal minimum wages,” with eight more response options in between. In the analysis, we categorized the variable into “less than 168 US dollars” and “more than 168 US dollars.”

#### Life course SEP

We developed a combined indicator for SEP across childhood, adolescence, and adulthood. This indicator addressed the multi-dimensionality of SEP by accounting for different dimensions of SEP, such as occupation, income of participant, education, and housing conditions across different epochs. The construction of this indicator was carried out in two phases. In phase 1, we assigned scores for each of these socioeconomic indicators, and in phase 2, we summed these scores. The final score ranged from 0 (worse) to 13 (good). Occupation scores ranged from 1 to 4, with 4 being those with mother employed in childhood and adolescence and participant employed in adulthood, 2 and 3 being different combinations of mother’s employment status during childhood and adolescence and participant occupation, and finally, 1 was assigned to those whose mother was unemployed in childhood and adolescence and the participant was unemployed. The income of the participant was used as a two-level indicator, with 1 being income ≤168 US dollars (below legal minimum wage) and 2 > 168 US dollars (above legal minimum wage), based on the survey response categorization. Education scores were created based on the mother’s education in childhood and adolescence, and they ranged from 1 to 3, with higher scores reflecting a higher level of education (high school or more). Finally, housing scores ranged from 1 to 2, with 2 being those who lived all their lives in cement-constructed homes (in Colombia, houses built with cement, compared to other materials, tend to be more expensive and durable). Continuous life course SEP indicator was categorized as low (<5), medium (5–7), and high (>8) based on its quartile distribution.

##### Hypertension control measure

To measure blood pressure, we used a digital blood pressure monitor whose brand is OMRON® BP710. We defined *control of hypertension* among those with hypertension as having diastolic and systolic blood pressure below 140 and 90 mmHg, respectively (42). At each year of assessment, hypertension control was determined.

### Statistical methods

Exploratory data analysis techniques such as histograms, QQ-Plots, box-plots, scatterplots, and basic descriptive (means, medians, and IQR) were used to assess the distribution of dependent and independent measures. Frequency distributions of the outcome will be used to identify the distribution of outcomes, which in turn will be conducive to determining the appropriate modeling strategies. In addition, these techniques will also be used to explore the most informative transformations of the covariates, confounders, and relevant predictors considered in the analysis. Extreme values were examined using the Tukey-Fences approach, and their removal from the analysis was determined. Missing patterns and rates were assessed. Since rates were < 5%, a complete case analysis was considered.

For Aim 1, we considered descriptive statistics for the life course SEP overall and by city. Comparisons were addressed using standardized differences, SD; if SD > 0.2, we claimed statistical differences across cities of a cumulative social indicator.

For our specific Aim 2 and related hypothesis, we used a generalized linear mixed model (GLMM) with random intercept, with normal distribution, identity link, and exchangeable correlation matrix. GLMM models account for the repeated nature of the outcome as well as the clustering of patients within institutions and institutions within cities. Given the low number of selected cities (3) and institutions, we will not focus on estimating these effects, but rather GLMM will allow us to use the Huber–White standard error estimates, also known as “robust standard error” or “sandwich variance” estimates. In the case of a linear model with a working independence variance structure, these are known as “heteroscedasticity consistent standard error” estimators. They are a popular alternative to the likelihood-based generalized linear mixed model, which is more sensitive to variance structure specification. Potential multi-collinearity between relevant predictors was assessed using variance inflation (VIF) analysis. No predictors had VIF > 10.

Using the second hypothesis of Aim 2 as an example, the proposed model can be written as


$$\begin{array}{l} \ln\left(\frac{p}{1-p}\right)=\beta_{0}+\beta_{1}*\text{Life}\;\text{course}\;SEP\;\text{score}+\beta_{2}*\text{Time}\\ \;+\beta_{3}*\text{LCSS}*\text{Time}+\text{Covariates} \end{array}$$

Where p = P (Hypertension Control = 1), the life course SEP score is a categorical measure, taking 1 for low SEP and 3 for high SEP. Time is a categorical variable, taking the values of 1, 2, and 3 for each time when hypertension was measured. Appropriately indicating the categorical nature of the predictors, we will obtain one less than levels parameter estimates. Thus, for life course SEP (using reference = 2), we expect to estimate 2 parameters ($\beta_{11,}\;\text{and}\;\beta_{13,})$, 2 for time, and 2 for the LCSP*time interaction. All models were adjusted for demographic variables such as age, gender, race/ethnicity, and years in the hypertension program.

## Results

There were 258 participants recruited at baseline. In Table 1, we present some demographic information regarding the sample stratified by epoch.

**Table 1:** Demographics of hypertensive patients assessed by epoch.

Variables	Time 1 (*N* = 258)	Time 2 (*N* = 148)^*^	Time 3 (*N* = 137)^*^
*Sex*			
Women	62.4%	57.4%	59.1%
*Mean age*	58(SD = 6.5)	63.1(SD = 6.6)	64.0(6.5)
*Adulthood variables*			
*Education*			
Elementary school	23.3%	–	–
High school	22.5%	–	–
Elementary school incomplete or no studies	18.6%	–	–
Technical or technological studies	15.1%	–	–
University degree	14.3%	–	–
Postgraduate degree	6.2%	–	–
*Employment status*			
Student	20.9%	–	–
Retired	20.2%	–	–
Housekeeper	18.6%	–	–
Employee and student	18.6%	–	–
Independent worker	14%	–	–
Employed	3.5%	–	–
Unemployed	1.6%	–	–
*Income levels*			
≤$168 USD	81.4%	–	–
>$168 USD	18.6%	–	–
*Ethnicity*			
Mixed race	39.1%	–	–
Black	27.1%	–	–
White	23.3%	–	–
Mulatto	3.9%	–	–
Indigenous	0.8%	–	–
Childhood and adolescence variables			
*Mother’s educational level*			
No studies	31.8%	–	–
Elementary school	39.1%	–	–
High school	12.8%	–	–
Technical studies	2.7%	–	–
Technological studies	0.4%	–	–
University degree	1.6%	–	–
Postgraduate degree	0.4%	–	–
*Mother’s occupation*			
Employee	16.7%	–	–
Independent worker	21.3%	–	–
Student	0.4%	-	–
Housekeeper	56.2%	–	–
Absent	1.9%	–	–
Passed away	3.5%	–	–
*House materials*			
Cement mainly	38.4%	–	–
Wood mainly	39.1%	–	–
Other	22.1%	–	–

^*^Time 2 and Time 3 are not presented on the table since sociodemographic indicators were measured only in Time 1.

Table 2 shows a comparison of sociodemographic characteristics of participants across the three waves. The main differences in the composition of the sample were when comparing time 1 and 2, as well as time 1 and 3, while it was more stable between time 2 and 3.

**Table 2:** Comparison of baseline characteristics of participants’ contribution to different times.

					Standardized differences		
Measures	Level	Time 1 (*N* = 258)	Time 2 (*N* = 148)	Time 3 (*N* = 137)	Time 1 vs. Time 2	Time 1 vs. Time 3	Time 2 vs. Time 3
Age, Mean (SD)		0.58 (6.5)	63.1 (6.5)	64.0 (6.5)	−0.015	0.000	0.015
Years in the program, Mean (SD)		10.3 (18.7)	10.3 (19.1)	10.5 (19.7)	−0.021	−0.052	−0.031
Sex, *N* (%)	Female	161 (62.4)	85 (57.4)	81 (59.1)	8.370	6.914	−3.448
Race ethnicity							
Mixed-White, *N* (%)		161 (62.4)	97 (65.5)	91 (66.4)	−5.322	−10.608	−1.863
Black-Mulatto, *N* (%)		80 (31)	41 (27.7)	37 (27)	5.889	11.066	1.570
Other, *N* (%)		17 (6.9)	10 (7.1)	9 (6.9)	−0.641	0.000	0.784

Table 3 shows the changes in the blood pressure of the participants of the study at the three time points assessed. These results show that patients had progressively lower systolic blood pressure and higher levels of hypertension control.

**Table 3:** Mean and standard deviations for blood pressure and control of hypertension in the three time points assessed.

Blood pressure	Time 1 (*N* = 258)	Time 2 (*N* = 200)	Time 3 (*N* = 137)
*Systolic*	125.6 (18.8)	120.5 (16.2)	119.7(17.2)
*Diastolic*	77.0 (12.6)	74.4 (11.4)	74.3(12.8)
Control of hypertension			
*Controlled*	78.8	76.5	81.3
*No controlled*	21.2	23.5	18.7

Table 4 shows models to address the first objective of the study, i.e., to assess the distribution of individual variables of life course SEP. Results show that none of the variables included in the model predict the control of hypertension in the sample.

**Table 4:** Aim 1 models: multivariate models estimates for control.

Predictor	Level	OR	CI	*p*-value
Participant income (ref = < 168 US dollars)	More than 168 US dollars	1.18	(0.64, 2.17)	0.5977
Participant education (ref = College or more)	<Elementary school	1.17	(0.79, 1.74)	0.4414
	High school	0.91	(0.64, 1.31)	0.6191
	More than High school	0.88	(0.71, 1.10)	0.2681
Mother education in participant adolescence (ref = More than High school)	< Elementary school	1.12	(0.75, 1.66)	0.5817
	High school	1.18	(0.80, 1.76)	0.4043
	More than High school	-	-	-
Participant occupation (ref = Employed)	Unemployed	0.96	(0.81, 1.12)	0.5897
	Housekeeper	0.93	(0.75, 1.14)	0.4734
	Retired	1.14	(0.84, 1.53)	0.4022
Mother’s occupation during participant’s childhood (ref = Housekeeper)	Unemployed	1	(0.81, 1.23)	0.9721
	Employed	-	-	-
Mother occupation during participant’s adolescence (ref = Housekeeper)	Unemployed	0.94	(0.77, 1.15)	0.5705
	Employed	-	-	-
House materials during childhood (ref = Wood)	Material	0.94	(0.74, 1.18)	0.5779
	Other	0.93	(0.70, 1.23)	0.6097
House materials during adolescence (ref = Wood)	Material	1.11	(0.88, 1.40)	0.3609
	Other	0.83	(0.60, 1.15)	0.2715

Models adjust for age, gender, race/ethnicity codes as white/mixed race (ref), black/mulatto, and other years in the HTA program.

Table 5 shows the estimates for hypertension control, with the different variables measured in the participant (excluding mother variables).

**Table 5:** Aim 2 model: mixed model estimates for control with different indicators used.

Predictor	Level	OR	CI	*p*-value
Participant income ref. = ≤168 US	>168 US	0.80	(0.35, 1.84)	0.6019
Occupation ref. = Always employed	Always Unemployed	0.96	(0.81, 1.12)	0.5897
	Different combination 2	0.71	(0.75, 1.14)	0.4734
	Different combination 3	1.14	(0.84, 1.53)	0.4022
Education ref. = < elementary school	High school	1.00	(0.8, 1.27)	0.9668
	More than high school	0.83	(0.61, 1.12)	0.2244
House materials ref. = wood	Cement	1.05	(0.85, 1.29)	0.6552

Models adjust for age, gender, race/ethnicity codes as white/mixed race (ref), black/mulatto, and other years in the hypertension program.

Results show that there is no effect of the variables in the model in the control of hypertension.

Table 6 shows models to address the second hypothesis of the last objective of the study, which sought to assess the association of the combined life course SEP and control of hypertension.

**Table 6:** Aim 2 model: mixed models estimates for control.

Effect		OR	95% CI	*p*-value
Lifelong SEP (ref = Transition)	Low stable	1.33	(1.03, 1.72)	0.0304
	High stable	1.21	(1.01, 1.46)	0.0447

Models adjust for age, gender, race/ethnicity codes as white/mixed race (ref), black/mulatto, and other years in the HTA program.

In these models, we found significant associations between the combined effect of life course SEP and the control of hypertension in both groups. In this sense, the group with a higher lifelong SEP and the group with a lower lifelong SEP showed better control of hypertension (OR = 1.21; *p* < 0.05; OR = 1.33; *p* < 0.05, respectively) compared to those whose SEP throughout life varied the most.

## Discussion

In this study, we aimed to have a deeper understanding of the role of life course SEP variables and hypertension control in a Colombian sample of individuals who were part of programs for the control of this chronic condition.

Descriptive results revealed that there were more women than men in this sample, with 58.1% of the participants having completed at least a high school level, which we can consider an educated sample considering the Colombian context [data from 2014 showed that only 44% of the population with ages between 24- and 65-years old population had an upper secondary education; (43)].

Regarding hypertension control, most of the sample was controlled, especially at Time 3. Our results show that patients had progressively lower systolic blood pressure and higher levels of hypertension control throughout time. This suggests that this sample had a high level of control when compared to other studies in Colombia, which reported much lower levels (7, 44, 45). These differences might be explained by the higher levels of adherence to the treatment found in another study with this same sample, particularly in terms of pharmacological treatment adherence (46). In addition, another possible explanation is that we have a sample of young people, and all of them have health insurance. Nevertheless, although most participants in our sample are controlled (78.8% for Time 1, 76.5% for Time 2, and 81.3% for Time 3), it is worth noting that they did not report good levels of quality of life, as pointed out by another study with this sample (47).

An important issue is that the programs for hypertension control used to have an emphasis on medications, neglecting health promotion and disease prevention interventions. These interventions are part of an integral approach to health. In such an approach, structural aspects of individuals’ lives need to be considered. The comprehension of the multidimensional processes that determine individual and collective health conditions (48) is essential to address the fundamental causes of the disease and improve the type of interventions available to the population.

Regarding the second objective of the study, we aimed to understand the role of different SEP variables throughout the lifetime (childhood, adolescence, and adulthood) in the hypertension control odds for three time points (T1, T2, and T3).

In the model using different SEP variables throughout the lifetime (see Table 5), results showed no association between single life course SEP indicators and hypertension control in the three time points analyzed. In this sense, our results are different from those reported in the literature. For example, in the Jackson Heart Study that assessed life course socioeconomic status and hypertension (37), results found that mothers’ education of at least a high school degree was associated with lower incident and prevalent hypertension ratio in adults. In our study, we did not find a relationship between the mother’s educational levels and the control of hypertension. In the same direction, the *mother’s occupation* was pointed out as a good predictor of health status in adulthood (38). In these tested models (SEP variables included as independent factors to predict hypertension control in adulthood), we did not find the same tendency in the data. It is important to note that in this study, we did not include paternal education and occupation because, in the literature reviewed, we found a prevalence of maternal variables to explain the health status of their child in adulthood. In addition, in the Colombian context, 69.7% of the households are female-headed, without the presence of a spouse, which means that women are very much in charge of educating and raising their children (49).

In a similar way, poor *housing conditions* have been pointed out as having a negative impact on health status (33, 50), but we could not prove this effect in this first model tested.

In our results, we did not find a relationship between adulthood SEP indicators and the control of hypertension. Although evidence shows that a high adult income and a higher occupation are inversely associated with hypertension management (51), our results were different from those reported in the literature. Nonetheless, it is important to stress that although our models that aimed to test the effect of SEP variables on hypertension control independently did not show statistically significant associations, when we tested the effect of the lifelong SEP (as a whole effect), we were able to find significant results. Those results are discussed below.

To inquire about the cumulative effects of SEP in hypertension control, we created a combined indicator of lifelong SEP (see Figure 1). Results showed that there is a differential effect of SEP over time in the control of hypertension. Those who kept a higher SEP during childhood, adolescence, and adulthood showed better control of hypertension in comparison with those whose SEP throughout life varied the most. Similarly, those with a lower lifelong SEP showed better control of hypertension in comparison to those whose SEP was changing throughout life. The previous results seem to support that having a changing SEP throughout life negatively affects the probability of hypertension control (see Table 6). This might be related to a less stable SEP, either because it increases or because it decreases over time. In sum, these results did not show evidence of SEP accumulative effects but did show evidence of downward and upward mobility effects.

While research on the impact of changes in SEP throughout the lifespan on health is relatively understudied (52), particularly in hypertension (53), it is well-established that individuals who maintain a relatively high SEP throughout their life course are more likely to experience better health, whereas those who consistently have a relatively low SEP or downward social mobility tend to experience negative health outcomes (52–54). In this regard, the aforementioned finding may seem somewhat counterintuitive. However, there are two potential explanations for it. First, studies have shown that social changes at the societal level are associated with higher personal stress because they disrupt lifepaths and might be linked to more potentially stressful circumstances (55). Changes in SEP might have a similar effect, leading to new stressful situations for individuals and resulting in a diminished sense of control, feelings of alienation, and experiences of discrimination throughout their lives. In fact, while downward social mobility can lead to stress and mental and physical disadvantages, upward mobile individuals can benefit from material wealth, prestige, power, and wellbeing. However, this may come at the expense of experiencing stressors hypothesized to lead to unhealthy behaviors and dysregulation of biological systems (56). If changes in both socioeconomic directions occur frequently, they might activate neuroendocrine mechanisms with implications for cardiometabolic health (53, 56), including increasing blood pressure (53, 57). In sum, changes in SEP throughout one’s life may challenge the individual’s homeostatic balance.

Second, in the Colombian context, a potential mechanism to explain this finding is that individuals who maintain a stable SEP throughout their lives may not have experienced changes in their health insurance regimen (contributory or subsidized). Since both regimens cover the treatment of hypertension, having a stable SEP could contribute to maintaining the affiliation to the same regimen, ensuring regular access to medication for controlling this chronic condition. In contrast, those who undergo changes in SEP during their life course are more likely to experience changes in their insurance affiliation, as they have to switch between regimens when their SEP changes (e.g., due to changes in occupational status or income). In this regard, a study in Colombia revealed high drop-out rates in a cohort of individuals affiliated with HEP, one of the explanations being a change in the occupational status of the participants (58). Thus, administrative barriers associated with the continuity of care and medications can lead to decreased control in individuals experiencing changes in their SEP and type of health insurance status.

This suggests that while the literature on social mobility and cardiovascular health supports the benefits of upward SEP through social policies aimed at reducing disadvantages across the lifespan (52, 53), having universal coverage in health insurance, as is almost the case in Colombia, might protect the most vulnerable population from a lack of access to healthcare. This, in turn, allows them to have similar results in controlling hypertension as those with a stable high SEP. Future studies have to rule out other mechanisms to explain this finding.

We would like to point out some of the limitations of this study. In this sample, we included an urban sample, leaving out an important part of the Colombian population that lives in rural settings; the previous would limit the adoption of a territorial approach in future decision-making, which is particularly important in a country such as Colombia, with a huge extension of rural and peasant territories. In addition, evidence shows a lower prevalence of hypertension in urban areas in Colombia (21%) in comparison with rural areas (34%) or mixed urban–rural areas (35%) (5), which points to a need to have a deeper understanding of rural territories in the future. Another limitation is that we did not include the father’s education and occupation during childhood in the analysis, and future studies should take this variable into account. In different cultures where the father is more present in raising children and contributing financially to the home, this variable becomes relevant. In addition, it is important to note that the study had a relatively small sample size, which could have an impact on the conclusions drawn. However, as we used a longitudinal design, this may potentially provide more power to the analysis performed.

## Conclusion

In this study, we aimed to address lifelong SEP variables and their relationship with hypertension control in Colombian adulthood, assessed at three different time points. This study contributes to the current body of research in the sense that there are relatively few studies exploring the relationship between social mobility variables and the control of hypertension. Most studies in the field of social mobility and health focus on looking at the relationship between SEP variables across the lifespan and their relationship with the prevalence and or incidence of cardiovascular disease, including hypertension [e.g., Leng et al. (37), Lopes et al. (59), and Glover et al. (53)]. In this study, we found that a stable SEP has a positive effect on hypertension control.

We contribute to this research by providing evidence that stable high or low SEP can experience benefits in hypertension control compared to those who undergo social mobility throughout their lives. While this may seem counterintuitive, it could be attributed to the stress associated with social mobility and the protective effects of health insurance among those with low, stable SEP in Colombia.

## Data availability statement

The raw data supporting the conclusions of this article will be made available by the authors, without undue reservation.

## Ethics statement

The studies involving humans were approved by Universidad de los Andes Ethics Committee (Act number 531 of 2015). The studies were conducted in accordance with the local legislation and institutional requirements. The participants provided their written informed consent to participate in this study.

## Author contributions

SB: Writing – original draft, Writing – review & editing. DL: Writing – original draft, Writing – review & editing. GM: Writing – original draft, Writing – review & editing. DA: Writing – original draft, Writing – review & editing.

## References

[1] World Health Organization (WHO). A global brief on hypertension. Silent killer, global public health crisis WHO Press (2013). Available at: https://iris.who.int/bitstream/handle/10665/79059/WHO_DCO_WHD_2013.2_eng.pdf?sequence=1

[2] Trevisol DJ Moreira LB Fuchs FD Fuchs SC. Health-related quality of life is worse in individuals with hypertension under drug treatment: results of population-based study. J Hum Hypertens. (2012) 26:374–80. doi: 10.1038/jhh.2011.48 21593782

[3] Xu X Rao Y Shi Z Liu L Chen C Zhao Y. Hypertension impact on health-related quality of life: a cross-sectional survey among middle-aged adults in Chongqing, China. Int J Hypertens. (2016) 2016:7404957. doi: 10.1155/2016/7404957, PMID: 27630771 PMC5005589

[4] NCD Risk Factor Collaboration (NCD-RisC). Worldwide trends in hypertension prevalence and progress in treatment and control from 1990 to 2019: a pooled analysis of 1201 population-representative studies with 104 million participants. Lancet. (2021) 398:957–80. doi: 10.1016/S0140-6736(21)01330-1, PMID: 34450083 PMC8446938

[5] Zurique-Sánchez M Zurique-Sánchez Camacho-López C Sánchez-Sanabria M Hernández-Hernández S. Prevalencia de hipertensión arterial en Colombia. Revisión sistemática y metaanálisis. Acta Méd Colombiana. (2019) 44:1–15. doi: 10.36104/amc.2019.1293

[6] Jiménez Soto A Cadena Gaona E Bermúdez Forero J Rosas Vargas L Benjumea Rincón M Poveda Rey N. Encuesta Nacional de la Situación Nutricional ENSIN 2015. Bogotá, Colombia: Instituto Colombiano de Bienestar Familiar (2019).

[7] Sánchez RA Ayala M Baglivo H Velázquez C Burlando G Kohlmann O. Latin American guidelines on hypertension. Latin American expert group. J Hypertens. (2009) 27:905–22. doi: 10.1097/HJH.0b013e32832aa6d2 19349909

[8] Leventhal AM Bello MS Galstyan E Higgins ST Barrington-Trimis JL. Association of Cumulative Socioeconomic and Health-Related Disadvantage with Disparities in smoking prevalence in the United States, 2008 to 2017. JAMA Intern Med. (2019) 179:777–85. doi: 10.1001/jamainternmed.2019.0192, PMID: 31009023 PMC6547249

[9] Shea S Lima J Diez-Roux A Jorgensen NW McClelland RL. Socioeconomic status and poor health outcome at 10 years of follow-up in the multi-ethnic study of atherosclerosis. PLoS One. (2016) 11:e0165651. doi: 10.1371/journal.pone.0165651, PMID: 27875557 PMC5119729

[10] Pollitt RA Rose KM Kaufman JS. Evaluating the evidence for models of life course socioeconomic factors and cardiovascular outcomes: a systematic review. BMC Public Health. (2005) 5:7. doi: 10.1186/1471-2458-5-7, PMID: 15661071 PMC548689

[11] Brown A Liang L Vassar S Merkin S Longstreth J Ovbiagele B. Neighborhood socioeconomic disadvantage and mortality after stroke. Neurology. (2013) 80:520–7. doi: 10.1212/WNL.0b013e31828154ae, PMID: 23284071 PMC3589286

[12] Stinghini S Carmeli C Jokela M Avendaño M Muenning P Guida F. Socioeconomic status and the 25 × 25 risk factors as determinants of premature mortality: a multicohort study and meta-analysis of 1·7 million men and women. Lancet. (2017) 389:1229–37. doi: 10.1016/S0140-6736(16)32380-7, PMID: 28159391 PMC5368415

[13] Höfelmann DA Gonzalez-Chica DA Peres KG Boing AF Peres MA. Chronic diseases and socioeconomic inequalities in quality of life among Brazilian adults: findings from a population-based study in southern Brazil. Eur J Public Health. (2018) 28:603–10. doi: 10.1093/eurpub/ckx224, PMID: 29294001

[14] Kim J-H Park E-C. Impact of socioeconomic status and subjective social class on overall and health-related quality of life. BMC Public Health. (2015) 15:783. doi: 10.1186/s12889-015-2014-9 26275823 PMC4536592

[15] Demakakos P Nazroo J Breeze E Marmot M. Socioeconomic status and health: the role of subjective social status. Soc Sci Med. (2008) 67:330–40. doi: 10.1016/j.socscimed.2008.03.038, PMID: 18440111 PMC2547480

[16] Richards L Maharani A Präg P. Subjective social status and allostatic load among older people in England: a longitudinal analysis. Soc Sci Med. (2023) 320:115749. doi: 10.1016/j.socscimed.2023.115749, PMID: 36738654

[17] Ferraro KF Shippee TP. Aging and cumulative inequality: how does inequality get under the skin. The Gerontologist. (2009) 49:333–43. doi: 10.1093/geront/gnp034, PMID: 19377044 PMC2721665

[18] Ferraro KF Shippee TP Schafer MH. A theory of age and the accumulation of inequality In: Bengtson VL Silverstein M Putney NM, editors. Handbook of theories of aging. 2nd ed. New York: Springer (2009). 413–33.

[19] Hamil-Luker J O’rand AM. Gender differences in the link between childhood socioeconomic conditions and heart attack risk in adulthood. Demography. (2007) 44:137–58. doi: 10.1353/dem.2007.0004, PMID: 17461340

[20] Non AL Román JC Gross CL Gilman SE Loucks EB Buka SL. Early childhood social disadvantage is associated with poor health behaviours in adulthood. Ann Hum Biol. (2016) 43:144–53. doi: 10.3109/03014460.2015.1136357, PMID: 26727037 PMC4977531

[21] White K. An introduction to the sociology of health and illness. 1st ed. London: SAGE Publications (2002).

[22] Mattei J Demissie S Falcon LM Ordovas JM Tucker K. Allostatic load is associated with chronic conditions in the Boston Puerto Rican health study. Soc Sci Med. (2010) 70:1988–96. doi: 10.1016/j.socscimed.2010.02.024, PMID: 20381934 PMC2907654

[23] Becher H Palm F Aigner A Safer A Urbanek C Buggle F. Socioeconomic Conditions in Childhood, Adolescence, and Adulthood and the Risk of Ischemic Stroke. Stroke. (2016) 47:173–179. doi: 10.1161/STROKEAHA.115.011523, PMID: 26604249

[24] Cohen S Janicki-Deverts D Chen E Matthews KA. Childhood socioeconomic status and adult health. Annals of the New York Academy of Sciences. (2010) 1186:37–55. doi: 10.1111/j.1749-6632.2009.05334.x, PMID: 20201867

[25] Smith GD Hart C. Life-course socioeconomic and behavioral influences on cardiovascular disease mortality: the collaborative study. American Journal of Public Health. (2002) 92:1295–1298. doi: 10.2105/AJPH.92.8.1295, PMID: 12144987 PMC1447233

[26] Monnat SM Chandler RF. Long term physical health consequences of adverse childhood experiences. Sociol Q. (2015) 56:723–52. doi: 10.1111/tsq.12107, PMID: 26500379 PMC4617302

[27] Nurius PS Fleming CM Brindle E. Life course pathways from adverse childhood experiences to adult physical health: A structural equation model. J Aging Health. (2019) 31:211–30. doi: 10.1177/0898264317726448 28845729 PMC12164666

[28] Galobardes B Smith GD Lynch JW. Systematic review of the influence of childhood socioeconomic circumstances on risk for cardiovascular disease in adulthood. Ann Epidemiol. (2006) 16:91–104. doi: 10.1016/j.annepidem.2005.06.053, PMID: 16257232

[29] Lawlor DA Ebrahim S Davey Smith G. Adverse socioeconomic position across the lifecourse increases coronary heart disease risk cumulatively: findings from the British women’s heart and health study. Journal of Epidemiology and Community Health. (2005) 59:785–793. doi: 10.1136/jech.2004.029991, PMID: 16100318 PMC1733124

[30] Kivimaki M Lawlor DA Smith GD Keltikangas-Jarvinen L Elovainio M Vahtera J. Early socioeconomic position and blood pressure in childhood and adulthood. The cardiovascular risk in young Finns study. Hypertension. (2005) 47:39–44. doi: 10.1161/01.HYP.0000196682.43723.8a, PMID: 16330678

[31] Kagura J Adair LS Pisa PT Griffiths PL Pettifor JM Norris SA. Association of socioeconomic status change between infancy and adolescence, and blood pressure, in south African young adults: birth to twenty cohort. BMJ Open. (2016) 6:e008805. doi: 10.1136/bmjopen-2015-008805, PMID: 27029771 PMC4823398

[32] Galobardes B Shaw M Lawlor DA Lynch JW Smith GD. Indicators of socioeconomic position. J Epidemiol Public Health. (2006) 60:7–12. doi: 10.1136/jech.2004.023531 PMC2465546 16361448

[33] Krieger J Higgins DL. Housing and health: time again for public health action. Am J Public Health. (2002) 92:758–68. doi: 10.2105/AJPH.92.5.758, PMID: 11988443 PMC1447157

[34] Case A Fertig A Paxson C. The lasting impact of childhood health and circumstance. J Health Econ. (2005) 24:365–89. doi: 10.1016/j.jhealeco.2004.09.008, PMID: 15721050

[35] Wamani H Tylleskär T Astrøm AN Tumwine JK Peterson S. Mothers’ education but not fathers’ education, household assets or land ownership is the best predictor of child health inequalities in rural Uganda. Int J Equity Health. (2004) 3:9. doi: 10.1186/1475-9276-3-9, PMID: 15482596 PMC529301

[36] Nepal AK. What matters more for child health: A father’s education or mother’s education? World Dev Perspect. (2018) 10:24–33. doi: 10.1016/j.wdp.2018.09.002

[37] Glover L Cain-Shields L Wyatt S Gebreab S Diez-Roux A Sims M. Life course socioeconomic status and hypertension in African American adults: the Jackson heart study. Am J Hypertens. (2020) 33:84–91. doi: 10.1093/ajh/hpz133, PMID: 31420642 PMC6931894

[38] Pinilla J Lopez-Valcarcel BG Urbanos-Garrido RM. Estimating direct effects of parental occupation on Spaniards’ health by birth cohort. BMC Public Health. (2017) 17:2–9. doi: 10.1186/s12889-016-3997-6, PMID: 28056954 PMC5217274

[39] Mills KT Stefanescu A He J. The global epidemiology of hypertension. Nat Rev Nephrol. (2020) 16:223–37. doi: 10.1038/s41581-019-0244-2, PMID: 32024986 PMC7998524

[40] Patton MQ. Qualitative Evaluation and Research Methods. (1990) Newbury Park (CA): SAGE

[41] Telles EE. Pigmentocracies: Ethnicity, Race, and Color in Latin America. (2014) Chapel Hill: The University of North Carolina Press.

[42] Whelton PK Carey RM Aronow WS Casey DE Collins KJ Dennison Himmelfarb C. Guideline for the prevention, detection, evaluation, and Management of High Blood Pressure in adults. J Am Coll Cardiol. (2018) 71:e127–248. doi: 10.1016/j.jacc.2017.11.006, PMID: 29146535

[43] OECD (2014). Colombia - Country Note - Education at a Glance 2014: OECD Indicators. Available at: http://www.oecd.org/education/Colombia_EAG2014_CountryNote_ENG.pdf

[44] Barrera L Gómez F Ortega-Lenis D Corchuelo Ojeda J Méndez F. Prevalence, awareness, treatment and control of high blood pressure in the elderly according to the ethnic group. Colombian survey. Colombia Méd. (2019) 50:115–27. doi: 10.25100/cm.v50i2.4124, PMID: 31607768 PMC6774579

[45] Londoño Agudelo E Pérez Ospina V Battaglioli T Taborda Pérez C Gómez-Arias R Van der Stuyft P. Gaps in hypertension care and control: a population-based study in low-income urban Medellin, Colombia. Trop Med Int Health. (2021) 26:895–907. doi: 10.1111/tmi.13599, PMID: 33938098 PMC8453502

[46] Quiroz S Agudelo DM Lucumi D Mentz G. Asociación entre marcadores de posición social y adherencia al tratamiento de la hipertensión arterial en Colombia. Revista Chilena de Salud Pública. (2020) 24:11–22. doi: 10.5354/0719-5281.2020.57581

[47] Barradas S Lucumi D Agudelo D Mentz G. Socioeconomic position and quality of life among Colombian hypertensive patients: the mediating effect of perceived stress. Health Psychol Open. (2021) 8:205510292199693–9. doi: 10.1177/2055102921996934 PMC7905733 33747537

[48] Breilh J. Critical epidemiology and the people’s health. UK: Oxford University Press (2021).

[49] Departamento Administrativo Nacional de Estadística (2022). Encuesta Nacional de Calidad de Vida (ENC). Available at: https://www.dane.gov.co/files/investigaciones/condiciones_vida/calidad_vida/2022/Boletin_Tecnico_ECV_2022.pdf

[50] Adebowale SA Morakinyo OM Ana GR. Housing materials as predictors of under-five mortality in Nigeria: evidence from 2013 demographic and health survey. BMC Pediatr. (2017) 17:30. doi: 10.1186/s12887-016-0742-3, PMID: 28103828 PMC5248529

[51] Nakagomi A Yasufuku Y Ueno T Kondo K. Social determinants of hypertension in high-income countries: A narrative literature review and future directions. Hypertens Res. (2022) 45:1575–81. doi: 10.1038/s41440-022-00972-7, PMID: 35859023 PMC9296364

[52] Barakat C Konstantinidis T. A review of the relationship between socioeconomic status change and health. Int J Environ Res Public Health. (2023) 20:6249. doi: 10.3390/ijerph20136249, PMID: 37444097 PMC10341459

[53] Lopes JAS Giatti L Griep RH Lopes AADS Matos SMA Chor D. Life course socioeconomic position, intergenerational social mobility, and hypertension incidence in ELSA-Brasil. Am J Hypertens. (2021) 34:801–9. doi: 10.1093/ajh/hpab029 33544821

[54] Dronavalli M Page A Sperandei S Uribe G Huckel Schneider C Eastwood J. Determinants and health outcomes of trajectories of social mobility in Australia. SSM Popul Health. (2023) 21:101312–36. doi: 10.1016/j.ssmph.2023.101336, PMID: 36660174 PMC9843487

[55] Moen P. The uneven stress of social change: disruptions, disparities, and mental health. Soc Ment Health. (2022) 12:85–98. doi: 10.1177/21568693221100171

[56] Chen E Brody GH Miller GE. What are the health consequences of upward mobility? Annu Rev Psychol. (2022) 73:599–628. doi: 10.1146/annurev-psych-033020-122814, PMID: 34579546 PMC10142907

[57] Guimarães JMN Griep RH Clarke PJ Fonseca MJM Barreto SM Giatti L. Intragenerational Social Mobility and Changes in Blood Pressure: Longitudinal Analysis From the ELSA-Brasil Study. American Journal of Hypertension. (2018) 31:672–678. doi: 10.1093/ajh/hpy026 29438464

[58] Prada-Rios SI. Traslados entre EPS en Colombia: ¿Qué dicen las historias laborales de cotizantes en cinco ciudades del país? Revista de Gerencia y Políticas de Salud. (2016) 15:176–92. doi: 10.11144/Javeriana.rgyps15-30.tecq

[59] Leng B Jin Y Li G Chen L Ji N. Socioeconomic status and hypertension: a meta-analysis. J Hypertens. (2015) 33:221–9. doi: 10.1097/HJH.0000000000000428 25479029

